# Associations between socioeconomic deprivation and witnessing family violence among Swedish adolescents: findings from a population-based school survey

**DOI:** 10.1177/14034948251365333

**Published:** 2025-08-26

**Authors:** Sanna Tiikkaja, Ylva Tindberg, Natalie Durbeej

**Affiliations:** 1 Social Medicine/CHAP, Department of Public Health and Caring Sciences, Uppsala University, Uppsala, Sweden; 2 Centre of Clinical Research Sörmland, Uppsala University, Eskilstuna, Sweden; 3 Department of Women’s and Children’s Health, Uppsala University, Uppsala, Sweden

**Keywords:** Economic stress, social vulnerability, multi-type victimisation, witnessing physical/sexual violence

## Abstract

**Aim:**

Exposure to family violence is a significant public health concern, linked to negative outcomes in both childhood and adulthood. This study investigates the prevalence of family violence witnessed by adolescents in Sweden and explores its association with socioeconomic factors.

**Methods:**

Data from the population-based survey Life and Health in Youth conducted in 2023 was used, including 3704 adolescents aged 15–18 years. It examines three forms of family violence: threats of violence, abusive language and physical/sexual violence.

**Results:**

The findings reveal that 19% of adolescents reported having witnessed at least one type of family violence, with girls reporting higher rates than boys. Socioeconomic deprivation, particularly perceived family economic stress, was strongly correlated with witnessing family violence. Adolescents who experienced economic stress were more often reporting exposure to threats of violence (adjusted odds ratio (adjOR) = 2.84) and multi-type victimisation (adjOR = 2.81). These results indicate that socioeconomic factors are important to consider when supporting adolescents in vulnerable situations.

**Conclusions:**

**One in five adolescents reported having witnessed any type of family violence. This study provides insights into the association between socioeconomic deprivation and adolescent exposure to family violence within the Swedish context. Adolescents with these experiences should be recognised by professionals in the health sector, welfare services and educational system due to their particularly vulnerable situation.**

## Introduction

Exposure to family violence constitutes a significant public health concern, encompassing various aggressive behaviours occurring between family members. The aggressive behaviours most often referred to are intimate partner violence, child abuse and aggression between siblings often occurring in the home environment [1]. Witnessing these types of violence is a harmful, stressful and often traumatic experience and considered a risk factor for multiple negative outcomes in both childhood and adulthood [2-4]. Research indicates that direct or witnessed abuse is reported by 10–40% of children and adolescents [3,5-8].

Further studies on family violence indicate that family violence correlates with several individual and family related factors. For instance, girls report witnessing family violence more frequently than boys [5,8,9]. Adolescents that have witnessed family violence are at increased risk of being violent themselves or to bully others [10,11]. Another risk factor for children being exposed to family violence is belonging to a family with a low socioeconomic status [8,12-14]. According to a review, the following socioeconomic deprivation factors are related to children witnessing family violence: single parenthood, low parental education and low income [5]. More specifically, associations between poor financial situation [8] or low perceived income status in the family [15] and witnessing family violence have been reported. Additionally, children of unemployed [15,16] or divorced parents [17] are at risk of witnessing this type of violence. The prevalence of witnessing multiple types of violence (multi-type victimisation) is greater among adolescents in families with a poor economic situation compared with families with a good economic situation [7,18]. Research also proposes that children of migrant parents may face greater risk of witnessing family violence than children without this background. This increased risk is associated with stressors of the migration and assimilation process, concerns about the economy and lack of social network [19,20].

In Sweden, since 1 July 2021, witnessing family violence during childhood has been regulated by law. This legislation states that exposing a child to witnessing of physical or psychological violence within close family or household relationships constitutes a criminal offence. These offences among family members include acts seen or heard by the child such as threats of violence, the use of offensive language or controlling behaviour and physical or sexual violence. Moreover, according to the Swedish Health Care Law children are obliged to receive both information, advice and support when having witnessed family violence. Based on our current understanding, the witnessing of these types of family violence among Swedish adolescents has not been previously studied, nor has previous research explored how socioeconomic factors are associated with these different types of violence.

## Aims

The present study aimed to (1) describe the prevalence of witnessing different types of family violence, including multi-type-victimisation, during childhood, and (2) explore the association between socioeconomic factors and having witnessed such violence among adolescents in Sweden.

## Material and methods

The data for this population-based study were obtained from the web-based survey, Life and Health in Youth (LHY), conducted between February and March 2023 by the Department of Public Health and Welfare in the County Council of Sörmland, in collaboration with schools in the county of Sörmland [21]. School personnel distributed a code for accessing the anonymous survey, which the adolescents answered in the classroom. The questionnaire consisted of 74 questions for students attending ninth grade (Y9) (15–16-years old) and 78 questions for those attending the second year of upper secondary school (Y2U) (17–18-years old). Adolescents and their parents received written information about the LHY beforehand, including details about participation being voluntary, the options to not answer questions or to exit the survey at any point and the use of data for public health epidemiology and related research. This information was also given via a video shown prior to filling out the questionnaire. A completed questionnaire was, therefore, regarded as the student’s informed consent to participate. According to Swedish law, parental approval is not required for participants above the age of 15 [22]. The Swedish Ethical Review Authority approved the study (Dnr 2023-06739-01). Data from the LHY surveys have been used in previous research regarding adolescent health [3,23].

A total of 4435 adolescents completed the questionnaire (response rate 71% for Y9 and 61% for Y2U). The survey questions regarding the experience of witnessing family violence was not answered by 401 adolescents, who were therefore excluded from the current study. In addition, 330 adolescents had incomplete data, whereof 165 lacked information on parental work status (PWS), 45 lacked information on country of origin and 120 did not identify themselves as either a boy or a girl. In total, 3704 adolescents were included in the study.

### Dependent variable

Our dependent variable was having witnessed family violence, which was measured using three distinct questions, ‘Have you experienced that an adult in your family has. . . (here, we mean having seen or heard different types of family violence) (1) threatened to use violence against someone else in your family? (2) used abusive words, oppression or been dominant or controlling towards someone else in your family? (3) used physical violence against someone else in your family, such as hitting, pushing, kicking or sexual violence?’. The response options were ‘no’, ‘yes, one time’, ‘yes, 2–5 times’ and ‘yes, more than 5 times’. The respective response alternatives were then categorised into ‘yes, have witnessed’ or ‘no, have not witnessed’ for each type of family violence. A new variable was created for adolescents having witnessed any of the three types of family violence, categorised as either ‘yes’ or ‘no’. Furthermore, a variable for multi-type victimisation of family violence was created, categorised as having witnessed ‘3 types’, ‘2 types’, ‘1–2 types’ and ‘not having witnessed any type of family violence’.

### Covariates

The background factors included in the study were gender, (‘girl’ or ‘boy’) grade (‘Y9’ or ‘Y2U’) and country of origin was classified as ‘Swedish’ (‘born in Sweden’ or ‘having at least one Swedish parent’) or ‘non-Swedish’ (‘born outside of Sweden’ or ‘having both parents born outside of Sweden’). Socioeconomic deprivation was measured using three questions. (1) ‘Living with both parents’ was measured by: ‘Whom do you live with?’ Responses were categorised as ‘yes’ (‘living with both parents who live together’) or ‘no’ (‘living with my mother’, ‘living with my father’, ‘living with my mother and her partner’, ‘living with my father and his partner’, ‘living in a family home’, ‘living with other caregiver’ or ‘other’). (2) ‘Parental work status’ (PWS) was measured using two questions: ‘What does your mother/caregiver do?’ and ‘What does your father/caregiver do?’ The response alternatives included: ‘working’, ‘studying’, ‘unemployed’, ‘on sick leave’, ‘other/I don’t know’ and ‘have no mother/caregiver, father/caregiver’. The answers from the two questions were combined, with students reporting both parents/caregivers as ‘working’ or ‘studying’ categorised as having two parents working (both PW). Students who reported ‘unemployed’, ‘on sick leave’, ‘other/I don’t know’ or ‘have no mother/caregiver, father/caregiver’ were categorised as having at least one parent who was not working (⩾1 PNW). (3) Adolescents’ perceived ‘economic stress’ was measured using the question: ‘Are you worried about your family’s economy?’ Responses were categorised as ‘yes’ (‘yes, quite worried’ or ‘yes, very worried’) or ‘no’ (‘not especially worried’ or ‘not worried at all’).

## Methods

### Statistical analyses

Descriptive analyses were carried out to describe the population and to report the prevalence of family violence by type, as well as multi-type victimisation of family violence. Correlations between background variables, socioeconomic factors and the various types of family violence were performed using Pearson Chi-square tests. Univariate logistic regressions were performed to assess associations between the socioeconomic variables (living with both parents, PWS and economic stress) and having witnessed any form of family violence, separately. The aim of this analysis was to investigate which socioeconomic variable had the strongest association with family violence, to use it as the independent variable in the forthcoming logistic regression analyses.

Next, logistic regressions were performed to assess associations between the socioeconomic deprivation variable with the strongest association with family violence and having witnessed specific types of violence, including threats of violence, abusive words and physical/sexual violence. The analyses were conducted using two models: the first was an unadjusted model, and the second was adjusted for gender, grade, country of origin, living with both parents and PWS. Lastly, a corresponding two-step modelling approach was performed to investigate associations between the socioeconomic variable having the strongest association with family violence and having witnessed multi-type victimisation. Multicollinearity between the independent variables was assessed using Spearman’s correlations, all of which were below 0.7 (data not shown). Odds ratios (ORs) with 95% confidence intervals (CIs) were estimated. *P* values < 0.05 were regarded as statistically significant. We used SPSS version 29 for the statistical analyses.

## Results

### Sample characteristics

The study population was evenly distributed by gender, with half identifying as girls and half as boys. The majority were in Y9 (58%), were born in Sweden (72%), lived with both parents (61%), had both PW (82%) and did not report economic stress (84%; data not shown).

The most common form of family violence reported was witnessing abusive words (17%). The reported proportions of witnessing physical/sexual violence (9%) and witnessing threats of violence (8%) were lower (Supplementary Table 1). Nearly one in five adolescents (*n* =715, 19%) reported having witnessed any form of family violence (Table I). The proportion of girls reporting having witnessed threats of violence, abusive words or physical/sexual violence was twice as high as boys. Having non-Swedish country of origin was significantly associated with having witnessed threats of violence between adults in the family but not with the other forms of family violence. The socioeconomic variables, not living with both parents, having one or more parents not employed and reporting economic stress, were all associated with having witnessed threats of violence, abusive words or physical/sexual violence, as well as having experienced any type of family violence (Table I). Repeated exposure to family violence was most often reported for having witnessed abusive words, with 7% of adolescents reporting this more than five times (Supplementary Table 1).

**Table I.** Distribution of reported experiences of different types of family violence among adolescents by background and socioeconomic factors (*N* = 3704).

	Have witnessed threats to use violence		Have witnessed abusive words		Have witnessed physical/ sexual violence		Have witnessed any type of family violence		Have not witnessed any type of family violence
	n = 297 (8)	*p* value	*n* = 624 (17)	*p* value	*n* = 343 (9)	*p* value	*n* = 715 (19)	*p* value	*n* = 2989 (81)
**Gender**		0.001		0.001		0.001		0.001	
Girl	190 (12)		428 (24)		217 (14)		477 (26)		1383 (74)
Boy	107 (6)		196 (11)		126 (7)		238 (13)		1606 (87)
**Grade**		0.530		0.798		0.851		0.639	
Y9	131(9)		364 (17)		200 (10)		420 (20)		1727 (80)
Y2U	166 (9)		260 (17)		143 (10)		295 (19)		1262 (81)
**Country of origin**		0.027		0.556		0.071		0.443	
Swedish	194 (8)		453 (18)		229 (9)		521 (20)		2135 (80)
Non-Swedish	103 (11)		171 (17)		114 (12)		194 (19)		854 (82)
**Living with both parents**		0.001		0.001		0.001		0.001	
Yes	93 (5)		263 (12)		112 (5)		304 (14)		1952 (87)
No	204 (16)		361 (26)		231 (18)		411 (28)		1037 (72)
**Parental work status**		0.001		0.001		0.001		0.001	
Both PW	175 (7)		451 (15)		214 (8)		514 (17)		2505 (83)
⩾1 PNW	122 (20)		173 (26)		129 (21)		201 (29)		484 (71)
**Economic stress**		0.001		0.001		0.001		0.001	
No	186 (7)		419 (14)		223 (8)		493 (16)		2630 (84)
Yes	111 (24)		205 (36)		110 (24)		222 (38)		359 (62)

The Chi-square tests are all calculated compared with the reference group of adolescents who have not witnessed any family violence. The proportions (%) are shown in brackets.

PW, parents working; PNW, parent not working; Y9, ninth grade; Y2U, upper second grade.

A total of 10% of adolescents had witnessed one type of family violence, and the proportions of adolescents who had witnessed two, (4%) and respective three (6%) types, were lower (Table II). All covariates, except for grade, were significantly associated with having witnessed one, two or three types of family violence.

**Table II.** Distribution of reported number of types of family violence among adolescents by background and socioeconomic factors (*N* = 3704).

	Witnessed 1 type of family violence	Witnessed 2 types of family violence	Witnessed 3 types of family violence	Have not witnessed any type of family violence	*p* value
	*n* = 370 (10)	*n* = 141 (4)	*n* = 204 (6)	*n* = 2989 (81)	
**Gender**					0.001
Girl	252 (14)	92 (5)	133 (7)	1383 (74)	
Boy	118 (6)	49 (3)	71 (4)	1606 (87)	
**Grade**					0.670
Y9	223 (10)	84 (4)	113 (5)	1727 (80)	
Y2U	147 (9)	57 (4)	91 (6)	1262 (81)	
**Country of origin**					0.001
Swedish	295 (11)	97 (4)	129 (5)	2135 (80)	
Non-Swedish	75 (7)	44 (4)	75 (7)	854 (82)	
**Living with both parents**					0.001
Yes	190 (8)	64 (3)	50 (2)	1952 (87)	
No	180 (12)	77 (5)	154 (11)	1037 (72)	
**Parental work status**					0.001
Both PW	301 (10)	100 (3)	113 (4)	2505 (83)	
⩾1 PNW	69 (10)	41 (6)	91 (13)	484 (71)	
**Economic stress**					0.001
No	273 (9)	95 (3)	125 (4)	2630 (84)	
Yes	97 (17)	46 (8)	79 (14)	359 (62)	

The Chi-square tests are all calculated compared to the reference group of adolescents that have not witnessed any family violence. The proportions (%) are shown in brackets.

PW, parents working; PNW, parent not working; Y9, ninth grade; Y2U, upper second grade.

### Associations between socioeconomic deprivation factors and having witnessed violence

When assessing the associations between the three socioeconomic variables and having witnessed any form of family violence in separate models, the strongest relation was found for reported economic stress (OR = 3.30; 95% CI 2.72–4.01) compared with not reporting this (data not shown). The corresponding associations between reporting any family violence and not living with both parents (OR = 2.55; 95% CI 2.16–3.01) and reporting more than one PNW (OR = 2.02; 95% CI 1.67–2.48) were lower (data not shown). Therefore, the forthcoming logistic regression analyses were performed using economic stress as the primary exposure variable.

Reported economic stress was associated with having witnessed threats of violence, abusive words or physical/sexual violence, as well as with having experienced any type of family violence (Table III, model 1). The strongest association, with an OR of 4.37, was found between reported economic stress and witnessing threats of violence, compared with those not reporting economic stress. In the second model, which included full adjustment for all covariates, the association between experiencing economic stress and having witnessed abusive words, threats of violence and physical/sexual abuse remained significant but with lower adjusted odds ratios (adjORs), ranging from 2.28 to 2.84.

**Table III.** Logistic regressions between economic stress and having witnessed threats of violence, abusive words, physical/sexual violence or any family violence.

		Have witnessed threats to use violence	Have witnessed abusive words	Have witnessed physical/sexual violence	Have witnessed any family violence
Model 1	Economic stress				
	No	Reference	Reference	Reference	Reference
	Yes	4.37 (3.37–5.67)	3.58 (2.93–4.38)	3.46 (2.27–4.45)	3.30 (2.72–4.01)
Model 2★	Economic stress				
	No	Reference	Reference	Reference	Reference
	Yes	2.84 (2.16–3.75)	2.72 (2.19–3.36)	2.28 (1.74–2.99)	2.50 (2.03–3.07)
	Living with both parents				
	Yes	Reference	Reference	Reference	Reference
	No	3.15 (1.61–2.83)	2.20 (1.82–2.66)	3.26 (2.43–4.02)	2.18 (1.82–2.61)
	Parental work status				
	Both PW	Reference	Reference	Reference	Reference
	⩾1 PNW	2.13 (1.61–2.83)	1.35 (1.08–1.70)	1.94 (1.48–2.54)	1.49 (1.15–1.78)

★ Adjusted for gender, grade, country of origin, living with both parents and parental work status. Odds ratios; 95% confidence intervals are shown in brackets.

PW, parents working; PNW, parent not working.

The association between reported economic stress and having witnessed family violence increased gradually with the number of types of violence witnessed, that is, one, two or three types, compared with those not reporting economic stress (Table IV, model 1). The highest OR of 4.63 was seen for having witnessed three types of family violence. When adjusting for other covariates, the adjORs decreased; however, all remained statistically significant, with the strongest association found for witnessing three types of family violence (adjOR 2.81; Table IV, model 2).

**Table IV.** Logistic regressions between economic stress and having witnessed one, two or three types of family violence.

		Have witnessed 1 type of family violence	Have witnessed 2 types of family violence	Have witnessed 3 types of family violence
Model 1	Economic stress			
	No	Reference	Reference	Reference
	Yes	2.60 (2.01–3.36)	3.55 (2.45–5.13)	4.63 (3.42–6.27)
Model 2★	Economic stress			
	No	Reference	Reference	Reference
	Yes	2.32 (1.79–3.70)	2.75 (1.87–4.04)	2.81 (2.03–3.89)
	Living with both parents			
	Yes	Reference	Reference	Reference
	No	1.67 (1.33–2.10)	1.91 (1.34–2.74)	4.41 (3.32–6.23)
	Parental work status			
	Both PW	Reference	Reference	Reference
	⩾1 PNW	0.98 (0.75–1.34)	1.46 (0.96–2.21)	2.24 (1.61–3.31)

★ Adjusted for gender, grade, country of origin, living with both parents and parental work status. Odds ratios; 95% confidence intervals are shown in brackets.

PW, parents working; PNW, parent not working.

## Discussion

This population-based school study, investigating the association between socioeconomic deprivation and self-reported experiences of having witnessed family violence, shows strong associations between economic stress and witnessing different types of family violence during childhood. Experience of witnessing family violence was common, reported by one in five participating adolescents. The most frequently reported form of family violence was witnessing abusive words (17%), followed by witnessing physical/sexual abuse and threats of violence.

We observed robust associations between socioeconomic deprivation among adolescents and experiences of witnessing family violence, including different types of violence and multi-type victimisation. The strongest associations, after adjustments, were found between witnessing threats of violence (OR = 2.84) and multi-type victimisation, that is, reporting all three types of family violence (OR = 2.81), respectively, for adolescents reporting economic stress compared with those not reporting it.

Our noted prevalence of self-reported witnessed family violence (19%) is considerably higher than the 11–14% reported in previous Swedish studies [3,7]. However, the survey questions on family violence in the latter studies were phrased differently and did not include sexual violence, in contrast to the present study.

Another possible explanation is that family violence may have increased over time. However, this seems unlikely, as previous findings suggest that family violence actually decreased by 2% between 2016 and 2021 [7]. Our results, which show a clear association between family violence and socioeconomic deprivation within the household, are consistent with previous studies [8,12,13,24].

We have shown associations between experiencing economic stress and adolescent witnessing family violence. The ORs for having economic stress and witnessing various forms of family violence were similar in magnitude, regardless of whether the violence involved threats of violence, abusive words, physical/sexual violence or any type of family violence in the adjusted models. The associations between economic stress and the number of types of family violence witnessed were also of the same magnitude, however, indicating a dose–response relationship, with the strongest association observed between economic stress and multi-type victimisation.

Previous research has shown that women reporting problems with their household income also report higher rates of family violence, with a prevalence of up to 17% among women in the lowest economic group [24]. Similarly, in the present study, we found that girls were twice as likely to report having witnessed family violence of all types, regardless of the specific type of family violence or multi-type victimisation. Further investigations are needed to determine whether this suggests that girls are more vulnerable to witnessing family violence than boys [5,8,9] or whether they are more exposed due to spending more time at home. As far as we know, this is the first study to examine the link between socioeconomic deprivation in adolescence and the experience of witnessing family violence in a Swedish context. The findings are important for understanding the baseline of family violence, especially in light of recent changes to legislation. In Sweden, a tightening of the law against child abuse, which had been in place for more than 40 years, was extended in 2021 to include witnessing or hearing family violence. Understanding the association between socioeconomic deprivation and witnessed family violence is significant for all practitioners involved with children and adolescents who have experienced or are at risk of child abuse. The Adolescent Psychosocial Assessment Methods are effective tools for engaging with teenagers in a trustworthy manner [25], focusing on the assessment of the Home environment, Education and employment, Eating, peer-related Activities, Drugs, Sexuality, Suicide/depression and Safety from injury and violence (HEEADSSS). This method is recommended for professionals to address various challenges encountered by this age group.

## Strengths and limitations

One of the strengths of this study is its population-based design, which included over 3700 Swedish adolescents. The LHY survey in 2023 had a high response rate (71% Y9 and 61% Y2U) and covered adolescents from the whole county of Sörmland in Sweden, increasing the generalisability of the findings to other parts of Sweden. Another strength is that we assessed different types of family violence (threats of violence, abusive words and physical/sexual violence), which capture a range of aggressive behaviours among adult family members. It is well known that socioeconomic deprivation and economic stress are significant stressors in parents’ everyday lives.

A limitation of this study is its cross-sectional design. Thus, no causality can be established from the results. Additionally, self-reports can be considered a limitation, given the risk of under-reporting exposure to family violence. A total of 16% of eligible study participants were excluded due to missing data. These excluded students lacking information on having witnessed family violence, PWS or country of origin. It is also possible that students who had witnessed family violence did not attend school on the day of the survey. These circumstances might imply an underestimation of the relationship between economic stress and witnessing family violence. Another limitation is that we did not have information regarding the identity of the perpetrator or victim in relation to the family violence.

## Conclusions

One in five adolescents reported having witnessed any type of family violence. Socioeconomic deprivation is associated with witnessing family violence among adolescents.

Adolescents with these experiences should be recognised by professionals in healthcare, social services and schools due to their particularly vulnerable situation.

## Supplemental Material

sj-docx-1-sjp-10.1177_14034948251365333 – Supplemental material for Associations between socioeconomic deprivation and witnessing family violence among Swedish adolescents: findings from a population-based school survey Supplemental material, sj-docx-1-sjp-10.1177_14034948251365333 for Associations between socioeconomic deprivation and witnessing family violence among Swedish adolescents: findings from a population-based school survey by Sanna Tiikkaja, Ylva Tindberg and Natalie Durbeej in Scandinavian Journal of Public Health
