# Supplementary material for: Associations between socioeconomic deprivation and witnessing family violence among Swedish adolescents: findings from a population-based school survey
Source: Scand J Public Health. 2025 Aug 26;53(6):650–7. doi: 10.1177/14034948251365333 (PMC12381381; doi:10.1177/14034948251365333)
Supplement: sj-docx-1-sjp-10.1177_14034948251365333 – Supplemental material for Associations between socioeconomic deprivation and witnessing family violence among Swedish adolescents: findings from a population-based school survey [file sj-docx-1-sjp-10.1177_14034948251365333.docx]

**Supplementary Table 1**. Distribution of adolescents having witnessed family violence, by type and number of events (n=3704).

|  | n (%) |
| --- | --- |
| Have witnessed threats to use violence |  |
| 1 time | 115 (3) |
| 2-5 times | 88 (2) |
| 5 + times | 94 (3) |
| No | 3407 (92) |
|  |  |
| Have witnessed abusive words |  |
| 1 time | 219 (6) |
| 2-5 times | 163 (4) |
| 5 + times | 242 (7) |
| No | 3080 (83) |
|  |  |
| Have witnessed physical/sexual violence |  |
| 1 time | 125 (3) |
| 2-5 times | 102 (3) |
| 5+ times | 116 (3) |
| No | 3361 (90) |
